# Feasibility and Safety of Endoscopic Peroral Cholangioscopy in Surgically Altered Anatomy: A Systematic Review and Meta-Analysis

**DOI:** 10.3390/jcm15093514

**Published:** 2026-05-04

**Authors:** Noemi Gualandi, Pablo Cortegoso Valdivia, Giuliano Francesco Bonura, Tommaso Gabbani, Paola Soriani, Mauro Manno

**Affiliations:** 1 Gastroenterology and Digestive Endoscopy Unit, Azienda USL di Modena, 41012 Carpi, Italy; g.bonura@ausl.mo.it (G.F.B.); t.gabbani@ausl.mo.it (T.G.); m.manno@ausl.mo.it (M.M.); 2 Gastroenterology and Endoscopy Unit, University Hospital of Parma, 43126 Parma, Italy; cortegosopablo@yahoo.it; 3 Department of Clinical Research, University of Southern Denmark, 5230 Odense, Denmark; 4 Surgical Research Unit, Odense University Hospital og Svendborg Sygehus, 5700 Svendborg, Denmark

**Keywords:** peroral cholangioscopy, surgically altered anatomy, ERCP, biliary lithiasis, systematic review

## Abstract

**Background/Objectives**: Endoscopic retrograde cholangiopancreatography (ERCP) in patients with surgically altered anatomy (SAA) presents significant technical challenges due to altered bowel reconstructions. Endoscopic peroral cholangioscopy (POC) offers a less invasive alternative to percutaneous or transmural techniques, but robust evidence validating its performance in SAA is lacking. This systematic review and meta-analysis (SRMA) aims to evaluate the feasibility and safety of endoscopic POC as a primary strategy in SAA. **Methods**: A systematic search was performed on PubMed and Embase up to December 2025 for studies reporting cholangioscopic outcomes in SAA patients utilizing an endoscopic approach. The primary outcome was the cholangioscopic access rate (AR). Secondary outcomes included endoscopic success rate (SR), technical SR, and adverse events. Pooled rates were calculated using Generalized Linear Mixed Models (GLMM). **Results**: Eight studies comprising 469 patients were included. The pooled endoscopic SR was 86.7% (95% CI, 74.4–93.6%) but showed high heterogeneity (I^2^ = 79.9%), largely driven by the variation in anatomical reconstructions. Subgroup analysis revealed higher endoscopic SR in short-limb anatomies (86.5%) compared to long-limb configurations (76.2%). Conversely, once biliary cannulation was achieved, the procedure was highly reliable: the pooled cholangioscopic AR was 95.9% (95% CI, 78.7–99.3%), with no significant difference across anatomical subgroups. The pooled adverse event rate was 3.1% (95% CI, 1.3–6.8%), predominantly characterized by mild complications. **Conclusions**: Endoscopic POC is a feasible and safe technique for managing biliary disease in SAA. The procedure’s overall success appears to be strictly dependent on the ability to navigate the altered anatomy (endoscopic phase), whereas the cholangioscopic phase itself proves highly effective and reproducible once biliary access is secured.

## 1. Introduction

Endoscopic retrograde cholangiopancreatography (ERCP) is the standard procedure to gain access to the biliary system. While cholangiography provides only indirect visualization of the biliary tree, cholangioscopy overcomes this limitation, showcasing a fundamental role in specific clinical scenarios such as the treatment of difficult biliary lithiasis, where visual control enables electrohydraulic or laser lithotripsy, and the evaluation of indeterminate biliary strictures, where targeted biopsies significantly increase diagnostic accuracy [1,2,3].

However, access to the biliary tree can be technically challenging in patients with surgically altered anatomy (SAA), often hindering the standard ERCP approach. In recent years, the number of patients undergoing complex upper gastrointestinal (GI) surgery has significantly increased, due to population aging (higher life expectancy) and bariatric interventions [4,5,6,7]. Accordingly, the clinical burden of biliary disease in SAA patients is also rising, as biliary lithiasis is a common postoperative complication [8,9]. The “anatomical isolation” of the papilla (or the bilio-enteric, BE anastomosis) in specific surgical GI reconstruction has led endoscopists to develop anatomically tailored solutions: depending on the reconstruction type and on the limb length, biliary access may require specific endoscopes other than the traditional side-viewing duodenoscope, such as enteroscopes (either long or short) or colonoscopes, to navigate through the altered bowel pathway. Such heterogeneity in the SAA fundamentally alters the standard technical workflow, in terms of endoscopic delivery, compared to the routine outcomes in native anatomy.

Several solutions are currently available to gain access to the biliary tree in patients with SAA [10,11,12]. Endoscopic peroral cholangioscopy (POC) can be delivered via different approaches, such as the “mother-baby” system (dual operator), catheter-based systems (single operator, through the scope) or direct insertion (either with an enteroscope or with ultraslim scopes, directly or via overtube exchange) [13]. Current therapeutic algorithms and expert consensus suggest a stepwise approach, with workflows typically proposing endoscopic POC as the preferred first-line modality due to its less-invasive, physiological nature, reserving other options, such as percutaneous transhepatic cholangioscopy (PTCS), EUS-directed transgastric ERCP (EDGE) or laparoscopy-assisted (LA) ERCP, for cases of failed endoscopic access [14,15,16,17]. While the transhepatic technique is effective, it carries higher morbidity risks related to external drainage, and transmural techniques, though promising, introduce risks of stent migration and fistula persistence [18,19,20].

Nevertheless, robust evidence specifically validating the performance outcomes of endoscopic POC in SAA is lacking: most available data are derived from retrospective series, small single-center experiences, or heterogeneous cohorts, employing different populations, non-standardized outcome definitions, and inconsistent follow-up durations. This systematic review and meta-analysis (SRMA) aims to fortify the evidence supporting endoscopic POC as a primary strategy in the management algorithm of SAA patients, focusing on feasibility and safety.

## 2. Materials and Methods

### 2.1. Data Sources and Search Strategy

A systematic literature search was performed on PubMed/MEDLINE and Embase databases up to December 2025. The search strategy combined Medical Subject Headings (MeSH) and free-text terms related to “cholangioscopy” and “surgically altered anatomy”. A manual review of the reference list of included studies followed the electronic search. The complete search string is available in Appendix A. The protocol for this SRMA (Supplementary Material S1) was not previously registered. This decision was based on the initially exploratory nature of the literature search, which aimed to map the landscape of POC in SAA before formally defining the final quantitative outcomes.

### 2.2. Inclusion and Exclusion Criteria

Inclusion criteria were: (1) patients with SAA undergoing POC via an endoscopic approach (e.g., enteroscopy or standard endoscopy); (2) reporting extractable data on cholangioscopic outcomes. Exclusion criteria were: (1) procedures performed via percutaneous transhepatic access or transmural access, to ensure methodological homogeneity; (2) case reports, reviews, and studies with fewer than 10 patients; (3) studies not reporting specific outcomes for SAA subgroups; (4) studies published before the year 2000 (to reflect contemporary clinical practice and current technological standards, such as the introduction of device-assisted enteroscopy and modern cholangioscopy platforms).

### 2.3. Screening of References

After excluding duplicates, two authors independently screened references (N.G. and P.C.V.). Each author screened all references (title and abstract) according to the inclusion and exclusion criteria. In case of discrepancy, the article was included for full-text evaluation. This approach was repeated on included references with both authors assessing the full text. In case of discrepancy in the full-text evaluation, a third author (G.F.B.) would also evaluate the reference, and a consensus discussion among all three would determine the outcome.

### 2.4. Data Extraction

Data were extracted following the Preferred Reporting Items for Systematic Reviews and Meta-Analyses (PRISMA) (Supplementary Table S1) [21]. Data were independently extracted by two reviewers (N.G. and P.C.V.) into a standardized spreadsheet. Variables collected included: study design, specific anatomy type, endoscope type, cholangioscopy platform, and data regarding cholangioscopy outcomes and procedural safety. Anatomical reconstructions were stratified into five subgroups for analysis: (1) Pancreaticoduodenectomy (Whipple), (2) Roux-en-Y Gastric Bypass (RYGB, bariatric), (3) Roux-en-Y (RY-NB, non-bariatric), (4) Non-RY, and (5) Mixed.

### 2.5. Quality Assessment and Risk of Bias

The methodological quality of included observational studies was assessed by two independent reviewers (N.G. and P.C.V.) using an adapted version of the Newcastle-Ottawa Scale (NOS) [22], specifically modified for single-arm observational studies.

### 2.6. Definition of Outcomes

The primary outcome was the cholangioscopic access rate (AR), defined as the successful advancement of the cholangioscope into the target bile duct following biliary cannulation. This outcome was selected to specifically isolate and evaluate the feasibility of the cholangioscopic phase in SAA patients. Secondary outcomes included: (1) endoscopic success rate (SR); (2) cannulation SR; (3) technical SR; and (4) safety.

The certainty of evidence for the outcomes was assessed using the GRADE (Grading of Recommendations, Assessment, Development and Evaluation) approach. Given the observational nature of the included studies, the initial certainty was rated as “low” and subsequently downgraded in case of further limitations.

To address the potential selection bias inherent to the multi-step nature of these procedures, outcomes were analyzed using specific denominators restricted to the subset of patients relevant to each procedural phase:

#### 2.6.1. Endoscopic Phase

- Endoscopic SR: successful advancement of the endoscope to the papilla or BE anastomosis. Calculation: calculated exclusively using studies with an intention-to-treat (ITT) design regarding the endoscopic phase. Studies enrolling patients specifically for cholangioscopy after confirmed biliary access were excluded from this analysis.
- Cannulation SR: successful deep biliary cannulation among the subset of patients in whom the papilla or BE anastomosis was successfully reached.

#### 2.6.2. Cholangioscopic Phase

- Cholangioscopic AR: successful advancement of the cholangioscope into the target bile duct. Calculation: the denominator was defined as the total number of patients in whom cholangioscopy was attempted after successful cannulation.
- Technical SR: successful completion of the intended diagnostic or therapeutic maneuver. Calculation: calculated as a per-patient analysis. The denominator included all patients in whom cholangioscopy was attempted. Failures of access were counted as technical failures.

#### 2.6.3. Safety

- Adverse event rate: pooled rate of procedure-related (both endoscopy and cholangioscopy) adverse events. Calculation: The denominator was the total number of endoscopic procedures performed (per-procedure analysis).

### 2.7. Statistical Analysis

Given the presence of high proportions and sparse data, we used a Generalized Linear Mixed Model (GLMM) with logit transformation (PLOGIT) to minimize bias [23]. To account for the inherent clinical and methodological heterogeneity across observational studies involving SAA, a random-effects model (using Maximum Likelihood estimation) was applied as the primary analysis for all pooled outcomes, regardless of the I^2^ statistic. To address potential patient overlap among studies originating from the same institution with overlapping enrollment periods, a sensitivity analysis was performed by excluding redundant cohorts and retaining only the most comprehensive datasets to ensure the independence of the pooled observations.

Heterogeneity was quantified using the I^2^ statistic, Cochran’s Q test, and τ^2^. To investigate potential sources of heterogeneity, pre-specified subgroup analyses were performed based on the anatomical reconstruction type, assessing differences between subgroups via the Q-test. The primary analysis for endoscopic access and cannulation outcomes was conducted using an ITT approach to mitigate selection bias, excluding studies with “per-procedure” designs.

Assessment of publication bias using funnel plots and Egger’s regression test was planned but not performed, as the number of included studies was fewer than 10 for most outcomes, rendering these tests statistically underpowered according to Cochrane Handbook recommendations [24]. *p*-values < 0.05 were considered statistically significant. All statistical analyses were conducted using R statistical software (version 4.5.1; R Core Team 2025) utilizing the meta and tidyverse packages [25,26,27]. A large language model (Google Gemini 3 Pro) was used solely for language refinement and organizational support.

## 3. Results

### 3.1. Study Selection and Characteristics

**Figure 1:**
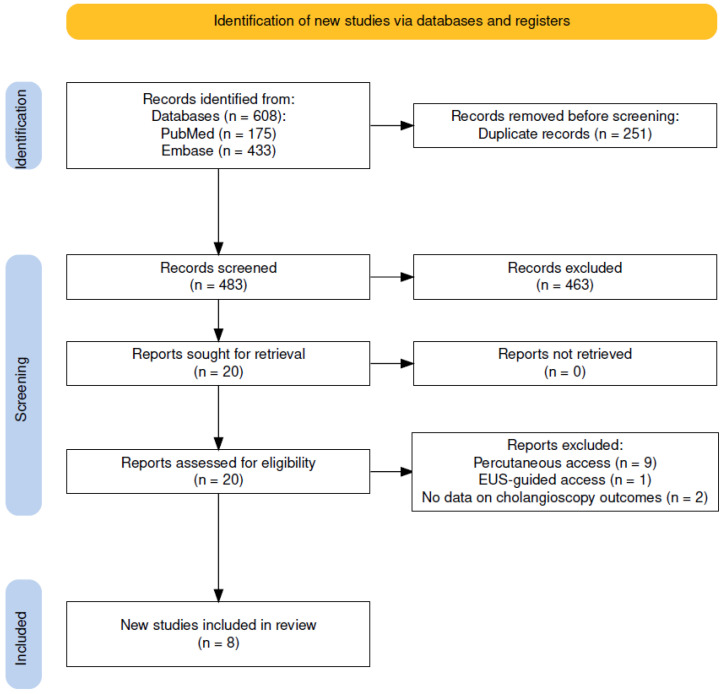
PRISMA flow chart.

Overall, 608 references were identified in the initial search. After exclusion of duplicates, abstract screening excluded 463 records, leaving 20 references for full-text reading. Eight (n = 8) studies (1 prospective, 7 retrospective), comprising 469 patients with SAA, were eventually included (Figure 1) [28,29,30,31,32,33,34,35]. The detailed characteristics of the included studies are summarized in (Table 1). Methodological quality assessment using the NOS yielded scores ranging from 5 to 8 (median: 7); while limitations were noted in the “Comparability” domain due to the predominant single-arm design, the included studies demonstrated high quality regarding patient selection and outcome ascertainment (Supplementary Table S2).

The anatomical distribution included 158 patients with RY-NB reconstruction, 63 with RYGB, 146 with pancreaticoduodenectomy, and 102 with non-RY or mixed anatomy.

The technical approach to gain biliary access varied across the included studies. Direct cholangioscopy with the enteroscope was performed in two studies [29,32]. In contrast, the overtube-exchange technique was the most frequent approach [30,31,33,35]; in these cases, the enteroscope served only to reach the target site and facilitate the placement of an overtube, through which an ultraslim scope was subsequently inserted. One study [34] utilized a catheter-based, through-the-scope system. Finally, one study [28] used a combination of the previously described techniques.

The primary indication for cholangioscopy was biliary lithiasis, accounting for 85.6% (n = 143) of the cholangioscopy cohort. A significant subset of these patients (60.8% of the stone cohort) presented with intrahepatic bile duct stones. Indeterminate biliary strictures represented 7.8% of cases.

**Figure 2:**
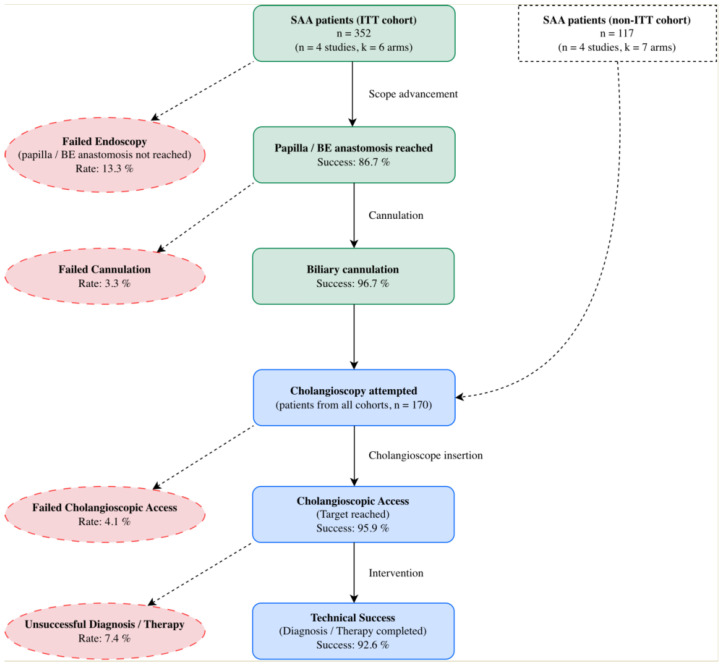
Flowchart of the study population and procedural success rates. Legend: BE, bilio-enteric; ITT, intention-to-treat; SAA, surgically altered anatomy.

The procedural workflow was analyzed in consecutive steps (Figure 2).

### 3.2. Endoscopic Phase

To evaluate the initial feasibility of the procedure, we analyzed the endoscopic SR exclusively in studies with an ITT design (k = 6, n = 352) [29,31,32,33]. The pooled endoscopic SR was 86.7% (95% confidence interval, CI, 74.4–93.6%) (Table 2). Substantial heterogeneity was observed for this outcome (I^2^ = 79.9%), reflecting the anatomical variability across cohorts. Among the subset of patients in whom the papilla or the BE anastomosis was successfully reached (n = 298), the biliary cannulation SR was 96.7% (95% CI, 85.3–99.3%; I^2^ = 8.6%) (Table 2).

### 3.3. Cholangioscopic Phase

**Figure 3:**
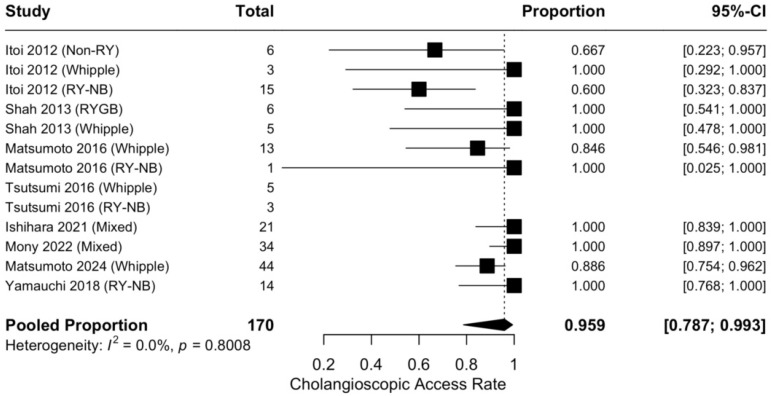
Forest plot of the pooled cholangioscopic access rate.

The pooled cholangioscopic AR, calculated in 170 patients across all included studies, was 95.9% (95% CI, 78.7–99.3%), with negligible heterogeneity (I^2^ = 0.0%) (Figure 3 and Table 2).

The overall technical SR was 92.6% (95% CI, 78.3–97.8%), with negligible heterogeneity (I^2^ = 0.0%) (Table 2). Notably, when calculated exclusively on patients in whom cholangioscopic access was achieved, the technical yield rose to 98.1% (152/155).

### 3.4. SAA Subgroup Analysis

To investigate the source of heterogeneity and the impact of anatomy on procedural success, we performed a stratified ITT subgroup analysis on 352 patients from studies providing granular anatomical data. We compared “long limb” configurations (i.e., RYGB; n = 63) [29] versus “short limb” reconstructions (i.e., Whipple, RY-NB, and non-RY; n = 289) [29,31,32,33].

**Figure 4:**
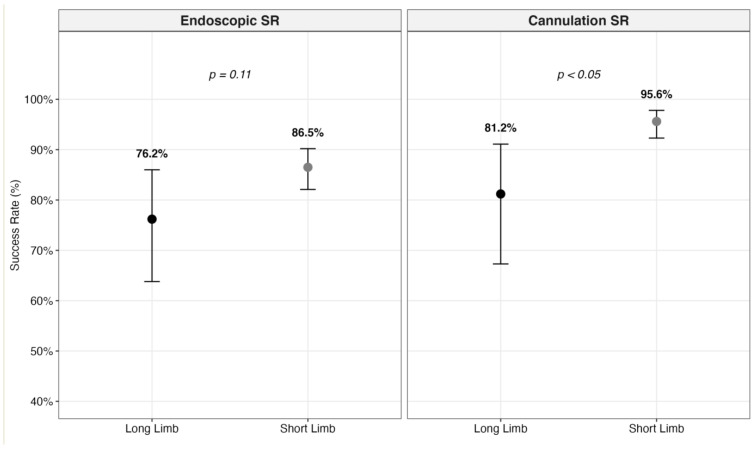
Comparison of pooled success rates according to limb length.

Granular analysis showed that endoscopic advancement to the papilla (or BE anastomosis) was successful in 86.5% of the short-limb group compared to 76.2% in the long-limb group, although this difference did not reach statistical significance (*p* = 0.11) (Figure 4 and Figure S1). Furthermore, biliary cannulation proved significantly more challenging in bariatric anatomy: the cannulation SR was 95.6% in the short-limb group compared to 81.2% in the long-limb group (*p* < 0.05) [Figure 4]. While limb length is crucial, the specific biliary anatomy (intact papilla or BE anastomosis) also significantly impacts procedural success [32]. However, a meta-analysis on this anatomical subset in the short-limb group was not feasible due to a lack of data stratification [31,33]. Conversely, once biliary access was secured, the cholangioscopic AR did not differ significantly across anatomical subgroups (*p* = 0.999) (Supplementary Figure S2): notably, 100% in the RYGB group (6/6) compared to 90.8% in the short-limb cohort (149/164).

### 3.5. Safety

**Figure 5:**
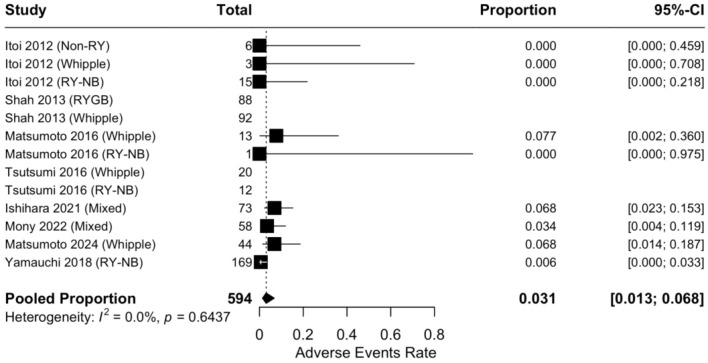
Forest plot of the pooled adverse events rate.

Safety data were analyzed for all endoscopic procedures (n = 594). The pooled rate of adverse events was 3.1% (95% CI, 1.3–6.8%, I^2^ = 0.0%) (Figure 5). Complications were predominantly mild (cholangitis, pancreatitis), with perforation rates remaining negligible (<1%). Mortality was limited to a single reported case [29].

### 3.6. Sensitivity Analysis

The sensitivity analysis conducted to account for potential patient overlap among the Okayama University Hospital group [30,31,33,35] confirmed the robustness of our findings. After excluding the earlier and potentially redundant cohorts from Matsumoto et al. (2016) [30] and Tsutsumi et al. (2017) [31], and retaining only the most comprehensive data from Ishihara et al. (2021) [33] and the subsequent cohort by Matsumoto et al. (2024) [35], the pooled estimates for all efficacy and safety outcomes remained consistent with the main analysis. Specifically, the estimates from the restricted dataset were: endoscopic SR 84.7% [95% CI, 71.0–92.7%] (original 86.7% [95% CI, 74.4–93.6%]), cannulation SR 94.7% [95% CI, 82.6–98.6%] (original 96.7% [95% CI, 85.3–99.3%]), cholangioscopic AR 97.6% [95% CI, 73.8–99.8%] (original 95.9% [95% CI, 78.7–99.3%]), technical SR 94.1% [95% CI, 75.5–98.8%] (original 92.6% [95% CI, 78.3–97.8%]), and adverse events 2.8% [95% CI, 1.1–6.8%] (original 3.1% [95% CI, 1.3–6.8%]). Crucially, the 95% CIs of these sensitivity estimates substantially overlapped with those of the primary analysis, confirming that the inclusion of the earlier cohorts did not introduce any significant shift in SRs or the safety profile.

As summarized in Table 2, the overall certainty of evidence assessed via the GRADE framework was rated as “very low” across all pooled outcomes. This was primarily driven by the retrospective, single-arm design of the included studies, along with suspected publication bias and imprecision for rare adverse events.

## 4. Discussion

The management of biliary disorders in patients with SAA represents a significant technical challenge in interventional endoscopy. The increasing prevalence of bariatric surgery and the improved survival of patients undergoing oncologic resections have led to a growing population requiring biliary access in the context of reconstructed GI tracts, and standard ERCP in these patients is limited by the inability to gain biliary access due to long surgical limbs, sharp angulations, and surgical adhesions. In this landscape, endoscopic POC represents a less invasive alternative compared to techniques such as PTCS, EDGE or LA-ERCP. It is important to note that recent evidence suggests that EDGE and LA-ERCP may offer superior technical SRs compared to enteroscopy-assisted approaches in long-limb reconstructions [20,36]. However, the true efficacy and safety of endoscopic POC have been difficult to isolate from the confounding variable of endoscopic reach.

The results of our SRMA suggest a clear dichotomy in the procedural workflow of endoscopic POC, which can be stratified into two subsequent, distinct phases. According to our analysis, the primary limitation of the procedure lies almost exclusively in the endoscopic phase: the pooled endoscopic SR of 86.7% showed high heterogeneity (I^2^ = 79.9%), reflecting the variability of surgical reconstructions. Specifically, SRs were notably higher in short-limb anatomies (94.1%) compared to long-limb configurations (76.2%), where post-surgical adhesions and limb length may significantly hinder scope advancement. The difficulty of this endoscopic phase was further highlighted by the SAMISEN-B multicenter registry on motorized spiral enteroscopy-assisted ERCP in SAA, reporting a therapeutic SR of only 54% and prematurely terminated due to safety concerns (overall adverse event rate of 14%, of which 7% severe) [37].

Conversely, the data demonstrate that once the anatomical hurdle is overcome and biliary cannulation is achieved, the performance of cholangioscopy is highly reliable, with a 95.9% pooled cholangioscopic AR, and no statistically significant difference across anatomical subgroups (*p* > 0.05). Furthermore, the technical yield rose to 98.1% when calculated exclusively on patients in whom cholangioscopic access was achieved. This suggests that the distinct technical challenges of navigating a cholangioscope through a long endoscope, such as friction, looping, and loss of force transmission, can be largely mitigated by current device iterations (e.g., dedicated digital cholangioscopes, ultraslim endoscopes). Effectively, the technical yield of POC in SAA, once biliary access is secured, appears comparable to that reported in patients with normal anatomy [38,39,40].

In terms of safety, the pooled adverse event rate of 3.1% is favorable when compared to alternative modalities. This is particularly relevant when contrasted with PTCS, EDGE or LA-ERCP, which literature suggests may carry higher rates of procedure-related complications [18,19,20]. While percutaneous approaches like SpyGlass™ (Boston Scientific, Marlborough, MA, USA) PTCS achieve clinical success rates up to 99% [17], our reported cholangioscopic AR of 95.9% represents a highly competitive balance between efficacy and invasiveness. The safety profile observed in our analysis was predominantly characterized by mild adverse events, likely related to intraductal irrigation, with a negligible rate of perforation despite the use of long endoscopes in altered bowel. Notably, this safety profile reflects the cumulative risk of the entire procedure (endoscopic and cholangioscopic).

Several limitations of this study must be acknowledged. First, our search strategy was limited to PubMed/MEDLINE and Embase, as trial registries and gray literature were not systematically searched, potentially resulting in the omission of ongoing studies or unpublished data; however, this approach was chosen to prioritize peer-reviewed evidence with complete data reporting. Second, the included studies were predominantly retrospective: this introduces an inherent selection bias, as patients with extremely unfavorable anatomy (e.g., ultra-long limb bypasses) may have been excluded a priori or referred directly to other techniques, potentially overestimating the endoscopic SR. Third, there is notable heterogeneity in the employed devices: the analysis aggregated data from different endoscopes (e.g., long and short enteroscopes, duodenoscopes, pediatric colonoscopes, gastroscopes) as well as different cholangioscopy platforms (catheter-based systems vs. ultraslim endoscopes); while our statistical model accounted for random effects, the specific mechanical properties of each combination could essentially impact the ease of cholangioscope insertion. Fourth, the definition of “technical success” varied considerably across the included studies: while we standardized this outcome to the resolution of the clinical indication, the disease complexity was not uniform across the cohort; a substantial portion of the treated patients had intrahepatic lithiasis, a condition that is technically more demanding than common bile duct stones, yet the high success rate suggests the procedure is robust even for this indication. Fifth, the SAA subgroup analysis is hampered by uneven distribution of data, limiting their generalizability; specifically, the long-limb cohort was derived from a single study, essentially dictating the subgroup performance metrics [29]. Sixth, the endoscopic phase analysis relied on ITT data where available only; therefore, the 86.7% endoscopic SR should be interpreted as a best-case estimate derived from expert centers; the reproducibility of these results in lower-volume centers, where expertise in endoscopy in SAA may be less consolidated, remains to be verified. Finally, the application of the GRADE framework underscored that the overall certainty of evidence for our findings is very low. This emphasizes that while endoscopic POC appears highly effective and safe, prospective, randomized trials are necessary to provide higher-quality evidence and assess the long-term durability of these procedures.

In conclusion, endoscopic POC appears to be a feasible and safe technique for the management of biliary disease in SAA. The procedure’s success is largely dictated by the ability to navigate SAAs, while the cholangioscopic phase itself proves to be highly effective and reproducible. Future prospective trials, incorporating long-term outcomes, such as stone recurrence and re-intervention rates, are warranted to further define and empower the role of endoscopic POC in the therapeutic workflow of SAA patients.

## Figures and Tables

**Table 1:** Characteristics of included studies.

Study (Author, Year) [Ref.]	Country	Design	Sample Size (n) *	Anatomy	Endoscopy Platform	Cholangioscope Delivery	Cholangioscopy Platform	Quality (NOS)
Itoi et al. (2012) [28]	International	Retrospective	24	Non-RY, RY-NB, Whipple	DAE, gastroscope, duodenoscope	Mixed (direct insertion, overtube exchange)	DAE, ultraslim gastroscope, standard gastroscope	6/9
Shah et al. (2013) [29]	USA	Retrospective	129	RYGB, Whipple, RY-NB	DAE	Direct insertion	DAE	8/9
Matsumoto et al. (2016) [30]	Japan	Retrospective	14	Whipple, RY-NB	DAE	Overtube exchange	Ultraslim gastroscope	5/9
Tsutsumi et al. (2017) [31]	Japan	Retrospective	32	Whipple, RY-NB	DAE	Overtube exchange	Ultraslim gastroscope	6/9
Yamauchi et al. (2018) [32]	Japan	Retrospective	118	RY-NB	DAE	Direct insertion	DAE	8/9
Ishihara et al. (2021) [33]	Japan	Retrospective	73	Non-RY, RY-NB, Whipple	DAE	Overtube exchange	Ultraslim gastroscope	7/9
Mony et al. (2022) [34]	International	Retrospective	35	Non-RY, RY-NB, Whipple	gastroscope, duodenoscope, colonoscope	TTS	SpyGlass™ DS	7/9
Matsumoto et al. (2024) [35]	Japan	Prospective	44	Whipple	DAE	Overtube exchange	Ultraslim gastroscope	7/9

Legend: DAE, device-assisted enteroscopy; RY, Roux-en-Y; RY-NB, Roux-en-Y (non-bariatric); RYGB, Roux-en-Y gastric bypass (bariatric); TTS, through-the-scope. Notes: * total baseline population of patients; denominators for specific outcomes are restricted to the subset of patients reaching each procedural phase (see forest plots).

**Table 2:** Pooled estimates of procedural outcomes and certainty of evidence (GRADE).

Outcome	No. of Studies (Arms)	Total Patients (n)	Pooled Rate % [95% CI]	Heterogeneity (I^2^)	Certainty of Evidence (GRADE)
Endoscopic SR *	4 (6)	352	86.7% [74.4–93.6]	79.9%	⊕◯◯◯ Very low ^1^
Cannulation SR °	4 (6)	298	96.7% [85.3–99.3]	8.6%	⊕◯◯◯ Very low ^1^
Cholangioscopic AR ^#^	8 (13)	170	95.9% [78.7–99.3]	0.0%	⊕◯◯◯ Very low ^1^
Technical SR	8 (13)	170	92.6% [78.3–97.8]	0.0%	⊕◯◯◯ Very low ^1^
Adverse Events ^§^	8 (13)	594 (procedures)	3.1% [1.3–6.8]	0.0%	⊕◯◯◯ Very low ^2^

Legend: AR, access rate; CI, confidence interval; SR, success rate. Notes: * calculated on intention-to-treat studies only; ° calculated among patients in whom the papilla/anastomosis was reached; ^#^ calculated among patients in whom cholangioscopy was attempted after successful cannulation (per-patient analysis).; ^§^ calculated per-procedure; ^1^ downgraded for residual risk of bias (due to single-arm designs and lack of comparability) and for suspected publication bias; ^2^ downgraded for residual risk of bias (due to single-arm designs and lack of comparability), suspected publication bias, and imprecision (rare events).

## Data Availability

The raw data supporting the conclusions of this article will be made available by the authors upon reasonable request.

## Supplementary Materials

The following supporting information can be downloaded at: https://www.mdpi.com/article/10.3390/jcm15093514/s1. Figure S1: Forest plot of the pooled endoscopic success rate (ITT) stratified by limb length; Figure S2: Forest plot of the pooled cholangioscopic access rate stratified by limb length; Table S1: PRISMA checklist; Table S2: Methodological Quality Assessment (Newcastle-Ottawa Scale); Supplementary Material S1: Study protocol.

## Abbreviations

The following abbreviations are used in this manuscript:

AR	Access rate
BE	Bilio-enteric
CI	Confidence interval
EDGE	EUS-directed transgastric ERCP
ERCP	Endoscopic retrograde cholangiopancreatography
GI	Gastrointestinal
GLMM	Generalized Linear Mixed Models
ITT	Intention-to-treat
LA	Laparoscopy-assisted
NOS	Newcastle-Ottawa Scale
POC	Per-oral cholangioscopy
PRISMA	Preferred Reporting Items for Systematic Reviews and Meta-Analyses
PTCS	Percutaneous transhepatic cholangioscopy
RY-NB	Roux-en-Y (non-bariatric)
RYGB	Roux-en-Y gastric bypass
SAA	Surgically altered anatomy
SR	Success rate
SRMA	Systematic review and meta-analysis

## Appendix A. Bibliographic Search

Bibliographic search strategies were performed on 7 December 2025 using the following search strategies:

Search string (Pubmed): (“Cholangioscopy”[Mesh] OR “Cholangioscopy”[Title/Abstract] OR “Cholangiopancreatoscopy”[Title/Abstract] OR “SpyGlass”[Title/Abstract] OR “Direct cholangioscopy”[Title/Abstract] OR “Video cholangioscopy”[Title/Abstract] OR “Peroral cholangioscopy”[Title/Abstract] OR “Percutaneous cholangioscopy”[Title/Abstract] OR “Transhepatic cholangioscopy”[Title/Abstract] OR PTCS[Title/Abstract]) AND (“Gastric Bypass”[Mesh] OR “Anastomosis, Roux-en-Y”[Mesh] OR “Gastrectomy”[Mesh] OR “Pancreaticoduodenectomy”[Mesh] OR “Roux-en-Y”[Title/Abstract] OR “RYGB”[Title/Abstract] OR “Billroth”[Title/Abstract] OR “Whipple”[Title/Abstract] OR “Altered anatomy”[Title/Abstract] OR “Surgically altered”[Title/Abstract] OR “Hepaticojejunostomy”[Title/Abstract] OR “Bowel reconstruction”[Title/Abstract] OR “Post-surgical anatomy”[Title/Abstract])—175 results.

Search string (Embase): ((exp cholangioscopy/ or cholangioscop*.ti,ab,kw. or SpyGlass.ti,ab,kw. or PTCS.ti,ab,kw.) and (exp “Roux en Y anastomosis”/or exp “stomach bypass”/or exp gastrectomy/or exp pancreatoduodenectomy/or (Roux-en-Y or RYGB or Billroth or Whipple or hepaticojejunostomy).ti,ab,kw. or ((altered or surgic*) adj3 anatom*).ti,ab,kw.)) not (exp animal/not exp human/) not (editorial or letter or note or conference review or short survey or erratum).pt. not “conference abstract”.pt.—433 results.
