# Supplementary material for: Feasibility and Safety of Endoscopic Peroral Cholangioscopy in Surgically Altered Anatomy: A Systematic Review and Meta-Analysis
Source: J Clin Med. 2026 May 4;15(9):3514. doi: 10.3390/jcm15093514 (PMC13163546; doi:10.3390/jcm15093514)
Supplement: Supplementary file 1 [file jcm-15-03514-s001.zip › Suppl.Material 1 rev.pdf]

Study protocol

"Feasibility and safety of endoscopic peroral cholangioscopy in surgically-altered anatomy: a systematic review and meta-analysis."

Background and Rationale

Endoscopic retrograde cholangiopancreatography (ERCP) in patients with surgically-altered anatomy (SAA) presents significant technical challenges. Peroral cholangioscopy (POC) has emerged as a valuable tool for direct visualization and therapeutic intervention in the biliary tree. However, evidence regarding its specific performance in the SAA population is unclear. This study aims to pool available data to provide robust estimates of its efficacy and safety.

Objectives

To evaluate the procedural success rates and the safety profile of various endoscopic POC techniques in patients with SAA.

Eligibility Criteria

- Population: Adult patients with SAA requiring biliary intervention.
- Intervention: Endoscopic POC
- Comparators: None
- Outcomes: cholangioscopic access rate (primary), endoscopic success rate, cannulation success rate, technical success rate, and safety.
- Study Design: Original prospective or retrospective observational studies, case series ($n \geq 10$).
- Exclusion Criteria: procedures performed via percutaneous transhepatic access or transmural access, to ensure methodological homogeneity; case reports, reviews, and studies with fewer than 10 patients; studies not reporting specific outcomes for SAA subgroups; studies published before the year 2000.

Information Sources and Search Strategy

See Appendix.

Study Selection and Data Extraction

Two reviewers independently screened titles and abstracts, followed by full-text review. Disagreements were resolved by a third senior reviewer. Data were independently extracted by two reviewers into a standardized spreadsheet. Variables collected included: study design, specific anatomy type, endoscope type, cholangioscopy platform, and data regarding cholangioscopy outcomes and procedural safety.

Risk of Bias Assessment

Quality assessment was performed by two independent reviewers using a modified Newcastle-Ottawa Scale (NOS) adapted for single-arm observational studies.

Statistical Analysis Plan

- Meta-analysis of proportions: performed using a General Linear Mixed Model (GLMM) with logit transformation (PLOGIT).
- Model Selection: A random-effects model was adopted as the primary analysis in the final analysis plan for all outcomes to account for clinical and methodological heterogeneity.
- Heterogeneity: Assessed using the I^2 statistic ($I^2 > 50\%$ indicating significant heterogeneity).
- Publication Bias: evaluated via funnel plots and Egger's test where applicable.
- Software: all analyses were conducted using R software (version 4.5.1) and the meta and tidyverse packages.

Certainty of Evidence

The certainty of evidence for each outcome was graded according to the GRADE framework as part of the final methodological refinement of the study to ensure the highest standard of evidence synthesis.
