# Supplementary material for: Feasibility and Safety of Endoscopic Peroral Cholangioscopy in Surgically Altered Anatomy: A Systematic Review and Meta-Analysis
Source: J Clin Med. 2026 May 4;15(9):3514. doi: 10.3390/jcm15093514 (PMC13163546; doi:10.3390/jcm15093514)
Supplement: Supplementary file 1 [file jcm-15-03514-s001.zip › Suppl.Table S1.pdf]

Supplementary Table S1. Methodological Quality Assessment (Newcastle-Ottawa Scale)

Study [ref]	*Year*	*Design*	*Selection (Max 4)*	*Comparability (Max 2)*	*Outcome (Max 3)*	*Total Score (Max 9)*	*Quality Rating*
Itoi et al. [28]	2012	Retrospective	3	0	3	**6**	**Moderate**
Shah et al. [29]	2013	Retrospective	4	1	3	**8**	**High**
Matsumoto et al. [30]	2016	Retrospective	3	0	2	**5**	**Moderate**
Tsutsumi et al. [31]	2017	Retrospective	3	0	3	**6**	**Moderate**
Yamauchi et al. [32]	2018	Retrospective	4	1	3	**8**	**High**
Ishihara et al. [33]	2021	Retrospective	4	0	3	**7**	**Good**
Mony et al. [34]	2022	Retrospective	4	0	3	**7**	**Good**
Matsumoto et al. [35]	2024	Prospective	4	0	3	**7**	**Good**

Note: *Newcastle-Ottawa Scale adapted for case series and single-arm studies. Modifications focus on cohort representativeness and anatomical stratification. Total scores: 7-9 (High/Good), 5-6 (Moderate), <5 (Low/Poor).*
