# Supplementary figures and images for: Feasibility and Safety of Endoscopic Peroral Cholangioscopy in Surgically Altered Anatomy: A Systematic Review and Meta-Analysis
Source: J Clin Med. 2026 May 4;15(9):3514. doi: 10.3390/jcm15093514 (PMC13163546; doi:10.3390/jcm15093514)

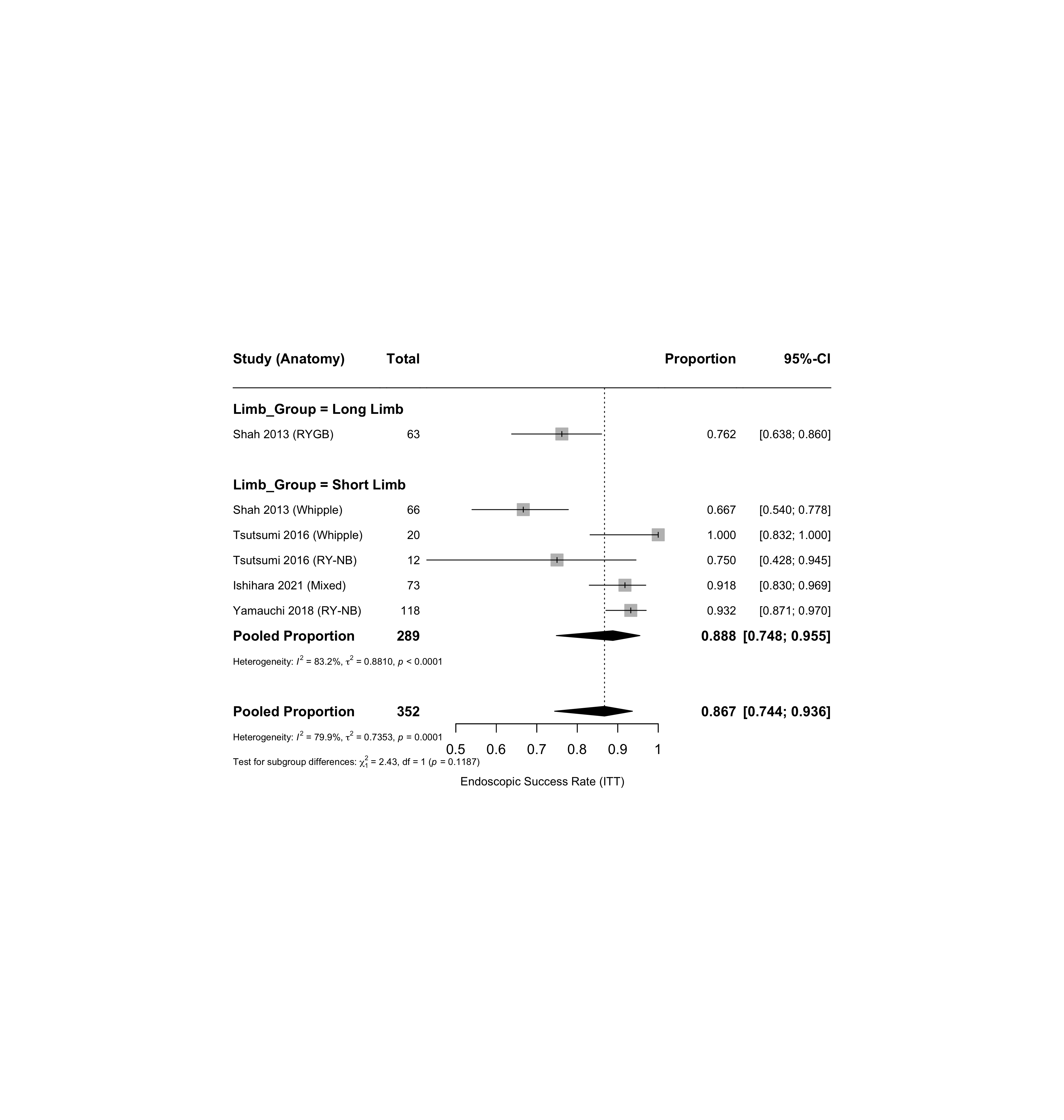

Supplement: Supplementary file 1 [file jcm-15-03514-s001.zip › Suppl.Figure S1.png]

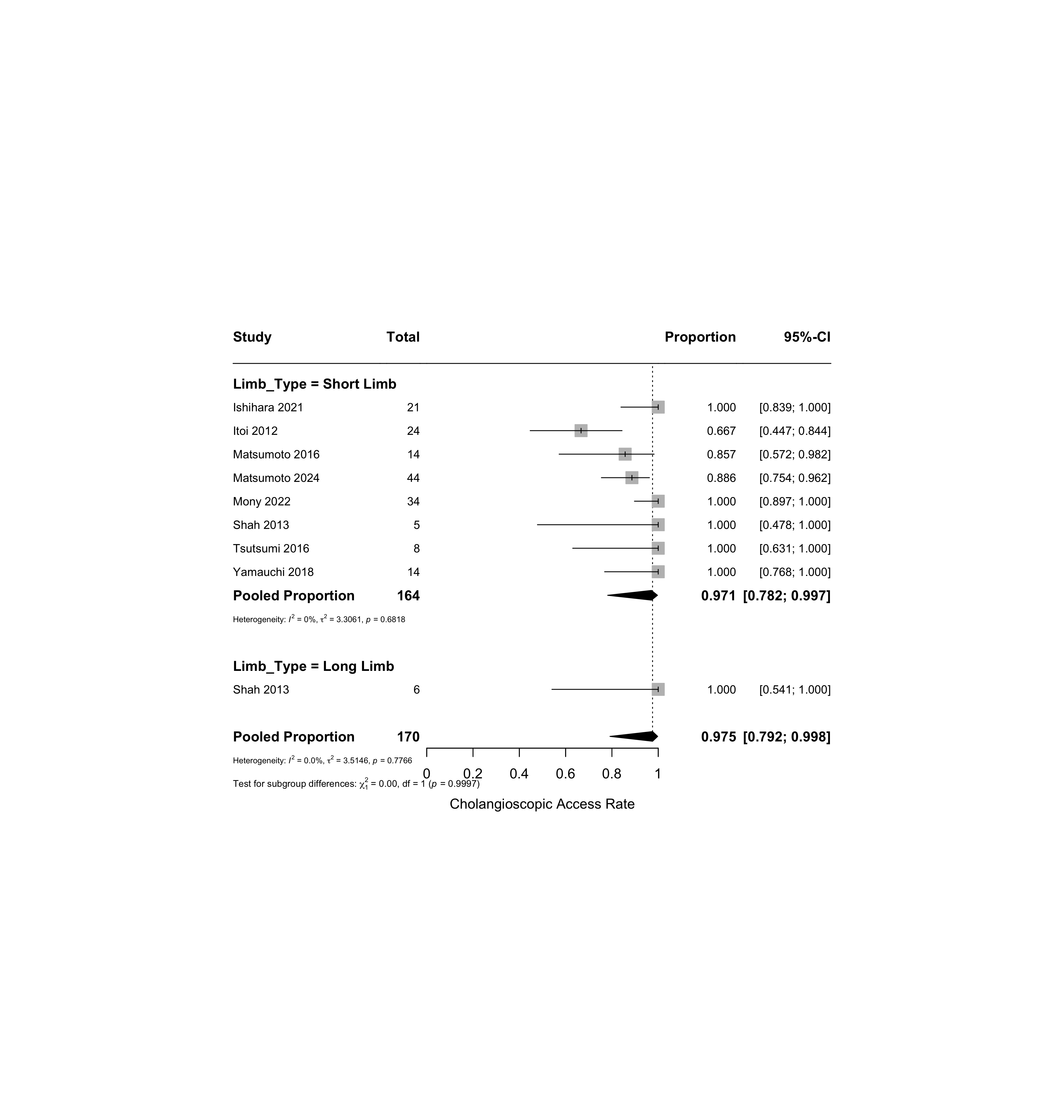

Supplement: Supplementary file 1 [file jcm-15-03514-s001.zip › Suppl.Figure S2.png]
